# Response to a cluster of Severe Acute Respiratory Syndrome Coronavirus 2 cases at a diagnostic laboratory

**DOI:** 10.4102/ajlm.v9i1.1307

**Published:** 2020-08-25

**Authors:** Christoffel J. Opperman, Gert J.K. Marais, Michelle Naidoo, Marvin Hsiao, Nazlee Samodien

**Affiliations:** 1 Division of Medical Microbiology, National Health Laboratory Service, University of Cape Town, Cape Town, South Africa; 2 Division of Medical Virology, National Health Laboratory Service, University of Cape Town and Groote Schuur Hospital, Cape Town, South Africa

**Keywords:** SARS-CoV-2, COVID-19, diagnostic laboratory, outbreak, occupational exposure

## Abstract

**Introduction:**

We report on the first documented cluster of Coronavirus Disease 2019 cases amongst diagnostic laboratory staff and outline some of the initial and ongoing steps that are being implemented to manage and prevent the spread of Severe Acute Respiratory Syndrome Coronavirus 2 (SARS-CoV-2) in our laboratory.

**Case presentation:**

On 24 April 2020, three staff members of a tertiary diagnostic laboratory in Groote Schuur Hospital, Cape Town, South Africa, tested positive for SARS-CoV-2. Within seven days, a further nine cases were identified, which suggested an outbreak and prompted a full investigation.

**Management and outcome:**

A multifaceted strategic approach was adopted to halt the spread of SARS-CoV-2 in our laboratory. Interventions focused on simultaneously establishing appropriate risk mitigation and stratification strategies through the upscaling of infection prevention and control measures, whilst minimising disruption to service delivery.

**Conclusion:**

Laboratory Coronavirus Disease 2019 outbreaks have the potential to cripple a laboratory’s testing capacity. Contingency planning and risk assessments should occur early, and interventions should be modified according to each laboratory’s available resources and infrastructure.

## Introduction

To our knowledge this is the first reported cluster of Coronavirus Disease 2019 (COVID-19) cases in a laboratory in South Africa. With transmission of Severe Acute Respiratory Syndrome Coronavirus 2 (SARS-CoV-2) firmly established in our communities, infection amongst laboratory staff and laboratory outbreaks pose a serious threat to both private and public pathology laboratories and have the potential to derail the already-strained pathology services. We outline some of the initial and ongoing steps that are being implemented in our laboratory to manage and prevent the spread of SARS-CoV-2.

## Ethical considerations

This work was done in accordance with all ethical standards for carrying out research, in an emergency situation, without direct contact with human or animal subjects. Institutional (National Health Laboratory Service) and departmental (Department Pathology, Division Microbiology,) approval was obtained.

## Case presentation

On 24 April 2020, three staff members at the National Health Laboratory Service situated within Groote Schuur Hospital, tested positive for SARS-CoV-2. Within seven days, a further nine cases were identified, which suggested an outbreak and prompted a full investigation. This resulted in a temporary closure of affected sections of the laboratory and a significant disruption to laboratory services in the subsequent week.

## Management and outcomes

### Outbreak investigation strategy

Once the initial cluster was identified, a task team comprising pathologists and managers from multiple pathology disciplines was established to lead an outbreak investigation, whilst simultaneously upscaling infection prevention and control measures. The completion of a line list and analysis of the descriptive epidemiology allowed for the identification of laboratory-specific risk factors and transmission ‘hotspots’.

### Screening for Severe Acute Respiratory Syndrome Coronavirus 2 infection amongst staff members

It is well described that asymptomatic and pre-symptomatic people can transmit SARS-CoV-2 effiently.^1^ Thus, three days after the three initial confirmed cases, samples were collected from all staff members over a one-week period, regardless of exposure. A designated swabbing station was set up in the laboratory and staff were informed to collect their own swabs for reverse transcriptase polymerase chain reaction (RT-PCR) testing. A video on the correct technique for self-sampling was circulated prior to sample collection. This further limited potential exposure of laboratory staff, whilst reducing strain on hospital testing sites, and facilitated a shorter time to result, allowing for the resumption of clinical diagnostic services. Currently, all laboratory staff are being screened daily with a temperature check and a simple symptom-based questionnaire to help identify cases early.^2^

### Response interventions and contact tracing

All staff that tested positive for SARS-CoV-2 by RT-PCR were instructed to self-isolate at home and to return to work only after two weeks, if there was a resolution of clinical symptoms. This was in accordance with the South African COVID-19 national guidelines.^3^ Contact tracing was carried out immediately for all laboratory personnel who tested positive, to contain the spread of the virus. The contact tracing was limited to laboratory workers. Expanded contact tracing, including family members, was conducted independently by the Western Cape Department of Health.

Asymptomatic staff with confirmed exposure to colleagues who tested positive by RT-PCR, were categorised as high-risk for COVID-19 according to national guidelines.^4^ These asymptomatic employees were given special leave to quarantine for seven days and were required to submit a nasopharyngeal or mid-turbinate swab on day 8 for RT-PCR testing. They were to return to work if their RT-PCR test was negative but had to continue daily symptom self-checks until day 14 post exposure.

In our laboratory, one staff member with known risk factors for severe COVID-19 passed away, whilst the remaining 11 (this includes the first three cases) qualified to return to work after 14 days; 10 of the 11 were mildly symptomatic and one was asymptomatic.^3,4^ Positive specimens have been stored with a view to conduct genomic sequencing and analysis. The resources to perform sequencing in real time were not available, although we did recognise that it would have been extremely helpful in deducing transmission dynamics.^5^

### Infection prevention and control measures reinforced in the laboratory

Laboratory infection prevention and control measures were communicated swiftly to all staff. The local pathology management team recommended several measures to combat intra-laboratory transmission of SARS-CoV-2. These included: wearing of appropriate masks for all staff as opposed to virology and specimen reception personnel only, frequent and thorough hand-washing and cleaning of surfaces, frequent sanitisation of hands with 70% alcohol-based solutions, correct usage of personal protective equipment in addition to masks in appropriate circumstances and practice of social distancing. To aid the implementation of the above-mentioned measures, masks and personal protective equipment were made available, and extra bottles of alcohol-based solutions were placed at the entry and exit points of the laboratory.^6^

### Risk mitigation strategies

A shift system was implemented where amenable, to increase social distancing and to avoid having to isolate or quarantine the whole workforce if virus transmission continued. The need to acutely reduce the size of the on-site workforce in the SARS-CoV-2 diagnostic laboratory had to be weighed carefully against the inevitable increase in test turn-around time this would cause and the resultant public health implications for the community it serves. Flexible solutions included staggering shifts and encouraging staff who could work from home to do so. In some departments, where feasible, senior staff over the age of 60 with risk factors^7^ for COVID-19 were advised not to return to work during the initial phase of the outbreak. It took much deliberation to determine which of the high-risk employees could stay at home, because it had to be balanced against the consequences on service delivery in the context of a pandemic. Extra personnel from other pathology departments within the National Health Laboratory Service, as well as from affiliated departments at the University of Cape Town, were trained as backup SARS-CoV-2 testing staff. Additional automated kit-based platforms requiring minimal molecular training to operate were also introduced. The purpose was to increase the operator pool and to avoid key-person dependency, which could potentially disrupt service delivery if any or all of the key persons were isolated or quarantined. Testing platforms included the Seegene Allplex™ 2019-nCoV assay (Seegene, Seoul, Republic of Korea) after in-house or NucliSENS® easyMag® nucleic acid extraction (bioMérieux, Marcy l’Étoile, France) and the Abbott RealTime SARS-CoV-2 assay (Abbott Laboratories, Abbott Park, Illinois, United States). Assay data were analysed according to the manufacturers’ instructions.

### Daily laboratory activities

All academic activities were suspended and only essential on-site meetings that did not violate social distancing measures were held. Areas where staff were less vigilant about infection prevention and control precautions, such as the tearoom, were identified and risks mitigated as far as possible. For the tea room, IPC measures included frequent reminders to practise social distancing, request that staff bring and clean their own crockery and cutlery, and limit on the number of staff members in the room at any given time to six people. In addition to the tearoom, high-touch areas, for example, communal telephones, scanners, keyboards, light switches and door handles, were also identified as high-risk areas. Therefore, more frequent surface cleaning and disinfection was instituted. Furthermore, carpooling by staff, especially from COVID-19 ‘hot spots’ in the Cape Metropole, was discouraged and management provided masks for those commuting to and from work via public transport.

### Maintaining service delivery

Sections of the laboratory (haematology and chemical pathology) needed to be closed briefly for decontamination during the outbreak. These samples were diverted to a neighbouring National Health Laboratory Service facility to avoid a breakdown in service delivery. However, the sudden transfer of large volume of samples to an understaffed and unprepared laboratory did present challenges that delayed result turn-around times. In another attempt for the laboratory to meet with the increased SARS-CoV-2 testing demands, non-priority tests were suspended unless suitably justified by the clinician.

### Communication

Staff were kept abreast of new COVID-19-related laboratory information through regular small group or Zoom (Zoom Video Communications, San Jose, California, United States) meetings. Laboratory section-specific communication strategies, such as WhatsApp (WhatsApp, Menlo Park, California, United States) groups, were implemented. Pathologists were also in regular telephonic contact with affected staff members who were in isolation to ensure that they had the necessary support during recovery. Transparency and collaboration were a prominent feature of our response. In particular, the laboratory worked with Groote Schuur Hospital and the provincial outbreak response team to minimise the service disruption and to ensure that the laboratory could be reopened both safely and timeously.

## Discussion

### Challenges and lessons learned

The lack of a guideline specific for management of a SARS-CoV-2 laboratory outbreak posed a significant challenge in mobilising a quick outbreak response plan. Although international guidelines were available and consulted, they were not applicable to our setting. The dynamic nature of the outbreak necessitated frequent revisions to staff scheduling rosters when certain employees had to be isolated or quarantined. This was not an easy task, especially because the skill levels amongst all staff were not equally matched. Another difficulty was ensuring the safety of staff from SARS-CoV-2, whilst travelling to and from work. Many relied on public transport and carpooling and had no alternative means of travel.

Lessons learned include the importance of early implementation of a symptom screening tool to detect cases earlier and to prevent spread within the laboratory. We realised the importance of developing contingency plans for any crisis causing laboratory closure in the future. Had there been an existing plan, referral of large numbers of samples would probably have occurred faster and more smoothly, without impacting test turn-around time negatively. This outbreak emphasised the need for skills transfer amongst staff to avoid key person dependency issues. We need to institute training programmes that will allow staff to fulfil multiple roles, thereby making it less likely that we jeopardise testing.

### Conclusion

A SARS-CoV-2 outbreak in a diagnostic laboratory can cripple its testing capacity, particularly one that is already under strain. A multifaceted strategic approach was adopted to halt the spread of SARS-CoV-2 in our laboratory, whilst minimising service delivery disruptions. Hopefully, our experiences serve to help other laboratories that find themselves in a similar situation. It is necessary for interventions to be modified based on each facility’s infrastructure and available resources. These recommendations should also be adjunctive to good laboratory practice principles.
